# Development of *Fewer Falls in MS*—An Online, Theory‐Based, Fall Prevention Self‐Management Programme for People With Multiple Sclerosis

**DOI:** 10.1111/hex.14154

**Published:** 2024-07-20

**Authors:** Susanna Tuvemo Johnson, Charlotte Ytterberg, Elizabeth Peterson, Sverker Johansson, Marie Kierkegaard, Kristina Gottberg, Maria Flink

**Affiliations:** ^1^ Department of Neurobiology, Care Science and Society Karolinska Institutet Stockholm Sweden; ^2^ Department of Women's and Children's Health Uppsala University Uppsala Sweden; ^3^ Women's Health and Allied Health Professionals Theme Karolinska University Hospital Stockholm Sweden; ^4^ Department of Occupational Therapy, College of Applied Health Sciences University of Illinois Chicago Chicago Illinois USA; ^5^ Academic Specialist Center Region Stockholm Stockholm Sweden

**Keywords:** action plan, complex intervention development, end‐user collaboration, group‐based intervention, multifactorial falls management

## Abstract

**Objective:**

The aim of this study was to describe the process used to develop a theory‐based, online fall prevention self‐management programme for ambulatory and non‐ambulatory people with multiple sclerosis (pwMS).

**Methods:**

The development process was guided by the Medical Research Council framework of complex interventions and began with a scoping review of the literature on self‐management of falls in pwMS. Subsequent phases of development were performed through iterative and concurrent processes and were informed by the perspectives of pwMS and healthcare professionals with MS expertise.

**Results:**

Through a systematic and iterative process in close collaboration with pwMS and healthcare professionals, a theory‐based online fall prevention self‐management programme, *Fewer Falls in MS*, for ambulatory and non‐ambulatory pwMS was developed. The programme is grounded in theory and pedagogical models and features utilization of action plans to address diverse influences on fall risks.

**Conclusions:**

A carefully operationalized definition of self‐management and an iterative co‐development process were essential to the creation of the *Fewer falls in MS* programme. Continuation of the co‐development process and collaboration with end users was needed to refine the programme.

**Patient or Public Contribution:**

PwMS and healthcare professionals were involved throughout the development process of the programme. The patient organization Neuro Sweden was contacted in the initial phase to discuss the relevance of a self‐management programme to prevent falls in MS. They supported the research group (all authors) in identification of and contact with pwMS with interest to participate. Three members of the research group (S.T.J., M.F. and C.Y.), that is, the operative group, met neuro Sweden and one pwMS to further discuss the relevance of a self‐management programme to prevent falls. To develop the process and content of the fall prevention programme, a co‐design process was performed together with pwMS and healthcare professionals. The results of the co‐design process are presented in this manuscript. In addition to participating in the co‐design process, pwMS and healthcare professionals provided feedback to the research group on programme process and content on several occasions during the subsequent programme development process. In a pretest (Beta version) of the programme, four pwMS acted as test subjects and provided additional feedback on the programme to the research group.

**Trial Registration:**

NCT04317716.

## 1 Introduction

Multiple sclerosis (MS) is a demyelinating and neurodegenerative disorder with a global prevalence of 36 per 100,000 inhabitants [1]. Due to the symptoms associated with MS, people with MS (pwMS) are at high risk for falls [2]. Approximately 70% of ambulatory pwMS fall each 6 months [3]. Those who have fallen have an 82% probability of experiencing a new fall within 6 months and a 56% probability of being injured in a fall [3].

Many MS symptoms are well‐known risk factors for falls. These symptoms include mobility limitations, impaired balance control and impaired cognition [4]. Decreased balance confidence and reduced fall self‐efficacy may also contribute to fall risk in pwMS [5]. Falls among pwMS are multifactorial as environmental, psychological, physiological and behavioural factors contribute to fall risk independently and through their interaction [6, 7].

The importance of taking the many and complex risk factors into consideration through fall prevention programmes for pwMS has been highlighted [8]. Despite this, most fall prevention programmes focus on addressing physical impairments and overlook environmental, psychological and behavioural influences on fall risk. Furthermore, the fall prevention intervention research involving pwMS has largely focussed on ambulatory individuals. Both online and face‐to‐face interventions for pwMS, who are full‐time wheelchair or scooter users (i.e., non‐ambulatory), show promising results but are rare [9, 10].

For people who are living with a chronic disease, the multifactorial influences on fall risks may be reduced through self‐management [11]. Self‐management can be defined as ‘the individual's ability to manage the symptoms, treatment, physical and psychosocial consequences and lifestyle changes intrinsic in living with chronic conditions’ [12]. This definition includes an understanding of factors associated with fall risks which is crucial to prevent falls during day‐to‐day activities.

Internationally, there is a growing interest in fall prevention interventions for pwMS that include attention to self‐management. To date, four pilot or feasibility studies [8, 13, 14, 15] and three randomized controlled studies (RCTs) [16, 17, 18] evaluating fall prevention interventions that include self‐management features have been published. While the extent to which self‐management components are defined and incorporated into the interventions described is variable [19], it is clear that self‐management is a promising approach to support pwMS in their efforts to prevent falls.

Consistent with the recommendations of O'Cathain et al. [20], who highlight the importance of involving end users in the development of complex interventions, MS fall prevention researchers are increasingly involving stakeholders. The involvement of stakeholders aims to develop interventions that reflect an empathetic understanding of users' fall prevention needs and the context in which fall prevention efforts are undertaken, to improve the likelihood of successful implementation and attainment of intervention outcomes sought (e.g., improved fall self‐efficacy and reduced fall incidence). For example, to inform the development of the *Better Balance* intervention, a fall‐prevention intervention for pwMS [21], Comber et al. conducted a telephone survey with pwMS to collect data about representative end users' preferences regarding intervention content and structure for fall prevention in pwMS. The co‐design process described by Carlgren et al. articulates how user's ideas and correlating needs can be identified and considered in the future development of the intervention [22].

The Medical Research Council (MRC) guidelines on development for complex interventions describe the importance of considering and documenting usage of existing interventions [23, 24]. Describing how an existing intervention or portion of an intervention has been used in a new context brings transparency to the intervention development process [25] and is an opportunity to build upon lessons learned from previous research. A thoughtful development process will also delineate the mechanisms of impact and theoretical underpinnings of the intervention [24]. For complex interventions, a well‐conducted development phase has better potential to result in positive outcomes for an intervention when evaluated, first for feasibility and later for effect, and thereby reducing research waste [20, 26].

Given these important trends in MS fall prevention research, the aim of this study was to describe the process used to develop the *Fewer Falls in MS* programme, a theory‐based, online fall prevention self‐management programme for ambulatory and non‐ambulatory pwMS. The programme was developed in Sweden in collaboration with a researcher in the United States of America.

## 2 Methods

### 2.1 Roles in the Development Process

Both pwMS and healthcare professionals were involved throughout the development process of the programme. The patient organization Neuro Sweden was contacted in the initial phase to discuss the relevance of a self‐management programme to prevent falls in MS. They supported the operative group (S.T.J., M.F., C.Y.) in identification of and contact with pwMS with interest to participate. The operative group included one social worker (M.F.) and two physiotherapists (S.T.J., C.Y.). Each had clinical and research expertise in MS, falls, co‐design and/or self‐management.

The operative group met Neuro Sweden and pwMS to further discuss the relevance of a self‐management programme to prevent falls. Even though representatives from Neuro Sweden and pwMS were not formally participants in the research group (all authors), they were throughout actively involved in all phases. Collectively, the research group were researchers who had expertise in MS care, fall prevention, rehabilitation, psychosocial support and self‐management. Members of the research group also had experience in developing and evaluating complex interventions designed to reduce fall risk, creating self‐management interventions, utilizing the design process for new health services interventions and engaging in person‐centred care. In addition to the research group, a group of co‐workers (pwMS and researchers in the Neuroepidemiology and health services research group at Karolinska Institutet in Sweden) continuously contributed to the development through discussions of hypotheses of outcome, content and format of the programme, methods for evaluation, emerging results and potential for future implementation.

### 2.2 Development Process Overview

**Figure 1:**
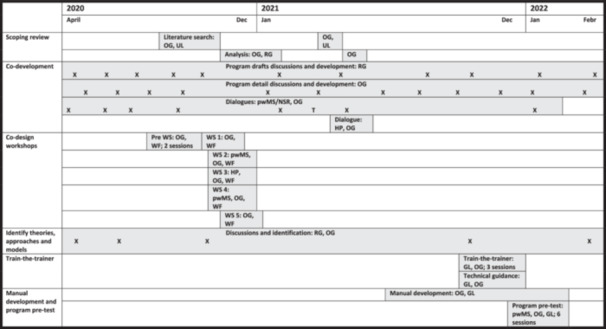
Overview of the methodological steps. Operative group (OG) (S.T.J., C.Y., M.F.), University Librarian (UL) Research group (RG), workshop via Zoom (WS), people with multiple sclerosis (pwMS), Neuro Sweden representatives (NSRs), workshop facilitator (WF), healthcare personnel (HP), group leaders (GL), meetings via Zoom or Teams (X) and T (telephone meeting).

After identifying the need for a self‐management intervention specifically designed for ambulatory and non‐ambulatory pwMS addressing a variety of individual risk factors, the research group was set up as an interdisciplinary team. The research group took the starting point for the development discussions in previously published literature, such as the article by Finlayson et al. [13], which inspired a potential programme format with group discussions and home exercises that encouraged participants to examine their own behaviour, attitudes, activity and environment to reduce risk of falling. Further steps taken to develop the intervention included a scoping review; co‐development; co‐design workshops; identification of guiding theories, approaches and models; train‐the‐trainer sessions and programme material development, and finally, a programme pretest (Figure 1). The development process was iterative in nature, with different phases occurring concurrently. An overview of the development process is presented in Figure 1. The development process was guided by the MRC guidelines [23, 24].

### 2.3 Scoping Review

A scoping review was conducted to investigate the extent and the scope of publications on self‐management of falls in pwMS and to identify how the concept of self‐management was defined and used in those publications [19]. The review was guided by the methodological guidance for scoping reviews [27, 28, 29]. Literatures published until July 2022 in the databases Ovid Medline, Cochrane, Web of Science and PsycInfo were included. The following specific inclusion criteria were applied: the publication included descriptions related to at least one self‐management task or at least one self‐management skill described by Lorig and Holman [30]; or, the authors described their work as self‐management; or, the publication included at least one outcome pertaining to improve self‐management skills, ability to engage in self‐management tasks or other behavioural change in relation to falls. Publications that did not include empirical data (e.g., trial registrations, conference abstracts, editorials, study protocols and grey literature) were excluded.

### 2.4 Co‐Development

The co‐development phase involved a series of meetings to discuss and develop programme content and format within the research group (all researchers): a small operative group that included members of the research group (S.T.J., M.F., C.Y.), pwMS, representatives from the patient organization Neuro Sweden and healthcare professionals. The co‐development phase began in April 2020 with meetings involving the operative group and representatives from Neuro Sweden.

Before each meeting, the operative group sent notes from the previous discussion to the research group, the pwMS or the healthcare professionals. In between the meetings, the operative group refined the programme, based on inputs from the meetings, and prepared the agenda for the next meeting.

After the co‐design workshops (see below), the format and content for the coming programme were discussed by the research group in several meetings. The discussions were based on the results of the five co‐design workshops: the emerging theory base for the programme (see below) and the fact that the then‐going COVID‐19 pandemic might affect the possibility of implementing the programme in physical premises.

### 2.5 Co‐Design Workshops

After initial planning meetings within the co‐developing phase, a structured co‐design process was initiated, based on design thinking [22], with the goal of strengthening the programme's quality and relevance to end users. The co‐design process utilized five 4‐h online workshops that occurred in December 2020. The online format was used due to the COVID‐19 pandemic. Each workshop involved the operative group and an external facilitator (innovation manager). In the first and the last workshops, respectively, the innovation manager and the operative group planned and summarized workshops two, three and four. The second and fourth workshops involved pwMS (six pwMS in each workshop), and the third workshop involved seven healthcare professionals. Twelve pwMS (10 women and two men) who could understand and communicate in Swedish, had the ability to use and access technical devices for online meetings (computer or tablet with internet access) and had experience with falls and/or near falls were recruited to the workshops. The pwMS were diverse with respect to time since diagnosis, disease severity and ambulatory capacity. The patient organization Neuro Sweden was involved in the recruitment of pwMS. Support on how to use the online meeting platform was provided by the members of the operative group to those in need of such support. The seven healthcare professionals (five women and two men) involved in the third workshop included two physiotherapists, two occupational therapists, one social worker, one registered nurse and one assistant nurse. Each had at least 9 years of experience in MS care and/or rehabilitation. The healthcare professionals were recruited through the research group's network.

The co‐design process was iterative with each workshop building upon the outcomes of the previous workshop(s). Each session began with an ice‐breaker activity and included both small‐group discussions in break‐out rooms and full‐group discussions to capture the individual statements and to take advantage of the group's dynamics. Online design tools such as storyboards and construct maps were used to get a common picture of the intervention sought and for modelling of a fall prevention intervention.

### 2.6 Identification of Guiding Theories, Approaches and Pedagogical Models

Concurrent with the co‐development phase and co‐design workshops, members of the research group worked together to identify guiding theories, approaches and models for the intervention. Because self‐management was a key focus in the development of the programme, the research group sought to define and operationalize self‐management and to investigate pedagogical models that would support development of the online intervention. When the guiding theories and approaches were identified, a mapping of the intervention was performed to clarify and describe the expected mechanisms of the intervention (Table 1).

**Table 1:** Mapping of the *Fewer Falls in MS* programme.

Session 1		
Purpose: To introduce the programme and strategies to effectively use online platforms		
Content	Social Cognitive Theory	Lorig and Holman tasks and skills
Welcome to the programme. Introduce ‘Check in’: How are you today? Short presentation of each of the participants. Review of the programme structure, schedule and its objectives, including action plans. The programme format, which includes online meetings and own practice and reflection between the sessions, is presented. Introduction of the digital tools Zoom and Canvas. Test the digital tools. Zoom: camera on/off, sound on/off, write text in the chat, to be divided into subgroups, ‘ask for help’ while in a breakout room Canvas: The platform organization and function are demonstrated. Participants are shown how to access PowerPoint presentations, videos, home assignments and chat. Approach and cooperation in the group, and joint agreement on group norms. Introduce the home assignments. Introduce ‘Check out’, i.e., reflection after this first meeting. Thoughts for the next session.	The participants learn via dialogue in the group, by trying and getting feedback on their results, in interaction with the environment (the platforms and others in the group) and from feedback on their behaviour, i.e., whether one succeeds or not = *reciprocal determinism*, and if you succeed = increased confidence in the ability to use the platforms = *self‐efficacy*	The participants have decided to attend the programme. To follow a programme and start changing behaviour, i.e., self‐care = *medical and role management.*

Home assignment to be completed after Session 1		
Purpose: Identify your own fall risks		
Content	Social Cognitive Theory	Lorig and Holman tasks and skills
Watch short, pre‐recorded videos of people with MS talking about their fall events and thoughts about them. Think about your own fall risk and take a picture with your mobile phone to illustrate a fall risk situation. Email the photo to the group leader. Test the asynchronous chat in Canvas.	To watch the films and reflect gives an opportunity for *observational learning.*	To reflect on one's own situation is the start of *problem‐solving.*

Session 2		
Purpose: To share experiences of falls/fall‐risk situations with each other, begin to explore influences on fall risk and prevention strategies		
Content	Social Cognitive Theory	Lorig and Holman tasks and skills
Check in. The session purpose is presented. Discussion on home assignment: participants share pictures and/or oral stories/experiences of fall risks/falls and discuss influences on fall risk leading to the fall. Discussion. Self‐care to prevent falls: identify risk, understand why it is a risk, set goals to reduce risk, identify activities, implement and evaluate. What strategies do the group members currently use to prevent falls? Introduction to the Session 2 home assignment. Check out: Reflection after this second meeting. Thoughts on this second session. Check the group's need for support in platform use, including content copying and sharing.	Listening to each other's stories and dialogue in the group is an opportunity for *observational learning* and potentially an opportunity for *reinforcement.*	To follow a programme and start changing behaviour, i.e., self‐care = *medical and role management. Problem‐solving* is started, in which problem definition is the first step.

Home assignment to be completed after Session 2		
Purpose: To formulate initial, individualized falls prevention goals		
Content	Social Cognitive Theory	Lorig and Holman tasks and skills
Watch a video about SMART goals. Reflect on activities that could lead to falls. Formulate and write down a fall prevention goal written in the context of an activity. *I will not fall when I … for the next … weeks*. Email your goal to the group leader	*Reciprocal determinism*: The interaction of person, environment and behaviour (to achieve goals).	To reflect on one's own fall‐risk situation is the start of *problem‐solving.*

Session 3		
Purpose: To set individual fall prevention goals and describe phases of a behaviour change process and create first action plans		
Content	Social Cognitive Theory	Lorig and Holman tasks and skills
Check in. The session purpose is presented. Homework follow‐up. Share and refine fall prevention goals. Lecture and discussion about: Goal setting and activities to reach goals, behaviour change, motivation, supportive and motivating factors. Barriers to behaviour change. Introduce the action plan template with goals, activities, etc. *What shall I have done within 2–4 weeks?* Obstacles on the way, and solutions. Own reflection: *How do I estimate my self‐confidence in performing the activities within the set plan?* If it seems unrealistic, revise your plan. Review of Home Assignment 3—To complete the action plan. Check out, reflection.	Interaction in the group contributes to *reciprocal determinism.* Watching and hearing about each other's goals is an opportunity for *observational learning* and is a potential for *reinforcement.* Estimation of self‐confidence relates to *outcome expectation.*	To follow a programme and start changing behaviour i.e., self‐care = *Medical and role management.* Based on knowledge about oneself and one's needs, make decisions about what exactly I need to do = *Decision‐making*, and then create a plan = *action planning.*

Home assignment to be completed after Session 3		
Purpose: Complete your own action plan		
Content	Social Cognitive Theory	Lorig and Holman tasks and skills
Watch three short videos about ‘*My action plan to prevent falls*’. Read three examples of completed action plans. Complete your own action plan based on the goal you set. Email the action plan to the group leader. If wanted, share thoughts on your action plan in the asynchronous chat.	Watching the videos and reading other examples of action plans is an opportunity for *observational learning*. *Outcome expectation* is examined via self‐grading in the action plan.	To complete an action plan implies *decision‐making, problem‐solving* and *action planning.*

Session 4		
Purpose: Refine the action plans		
Content	Social Cognitive Theory	Lorig and Holman tasks and skills
Check in. The session purpose is presented. Homework follow‐up/small group activity. Present, discuss, and refine action plans, point by point, and give each other feedback on the action plans. *What are your needs to prevent falls? How can this be integrated in your action plan?* Discussion (whole group): Findings from small group activity. Introduction to Home Assignment 4. Check out, reflection.	Feedback on action plans can promote *reinforcement.*	To follow a programme and start changing behaviour, i.e., self‐care = *Medical and role management.* Action plan discussions also have the potential to raise awareness of r*esource utilization* and *patient‐provider partnership*. For example, providers may be the focus of a goal or may be part of an action step.

Home assignment to be completed after Session 4		
Purpose: Implement action plan and evaluate effectiveness of fall prevention strategies during a valuated activity		
Content	Social Cognitive Theory	Lorig and Holman tasks and skills
Carry out the planned activity, using the fall prevention strategies identified in the action plan. Self‐evaluation. Was the goal of the action plan met? Rate 1–10. What went well? What did not go as planned? Why did the activity/and or fall prevention strategies not go as planned? Are there other fall prevention strategies that would have been more effective? Does the activity need to be changed to make it safer? Email the self‐evaluation to the group leader. If wanted, share thoughts on action plans in the chat.	Full or partial implementation of the activities/strategies, if successful builds *behaviour capability*, which leads to *self‐efficacy.* If the chat is used *reinforcement* might be achieved.	To carry out the activities implies *taking action*. Self‐evaluation supports *problem‐solving* and *decision‐making.*

Session 5		
Purpose: Follow‐up on the fall preventive activities. Reflect on own and others' expectations, which can affect the risk of falling. Refine action plan or create new action plan		
Content	Social Cognitive Theory	Lorig and Holman tasks and skills
Check in. The session purpose is presented. Whole‐group discussion: Homework findings. Evaluating the extent to which the goal of the action plan was met, and the effectiveness of fall prevention strategies during a valued activity. Revise and/or formulate a new action plan. Mini‐lecture and discussion: Balancing the body's ability to meet the expectations of the environment and one's own expectations, which can affect the risk of falling. Stop, reflect, ask for help or set limits based on your own needs. Introduction of Home Assignment 5. Check out, reflection.	Group discussion with feedback on completed actions leads to *reciprocal determinism.* Sharing experiences provides the opportunities for *observational learning,* and potentially is an opportunity for *reinforcement.*	To revise and formulate an action plan can be part of *Problem‐solving, decision‐making* and *self‐tailoring.*

Home assignment to be completed after Session 5		
Purpose: Implement action plan and evaluate effectiveness of fall preventive strategies		
Content	Social Cognitive Theory	Lorig and Holman tasks and skills
Carry out the planned fall preventive activities/strategies. Self‐evaluation, Rate 1–10*. What went well? How could you have done it any other way?* Email the self‐evaluation to the group leader. If wanted, share thoughts on action plans in the chat.	Internal feedback provides *reinforcement*. If one succeeds to some extent, *behavioural capability* is achieved, which leads to *self‐efficacy*.	To carry out the fall preventive activities implies *taking action* and to self‐evaluate supports *problem‐solving* and *decision‐making.*

Session 6		
Purpose: Start ‘closing the loop?’. Follow‐up on the action plan and discuss maintaining motivation		
Content	Social Cognitive Theory	Lorig and Holman tasks and skills
Check in. The session purpose is presented. Homework follow‐up/small group activity. The participants present and discuss their action plans and self‐evaluations. Mini‐lecture/discussion: How to maintain motivation: social support, diary reminders, rewards, and failures (as part of behaviour change) Individual activity: Write down your own tips on how to maintain motivation. Whole‐group discussion: Share strategies to maintain motivation to continue action planning/fall prevention efforts. Introduction of Home Assignment 6. Those of you who want to keep in contact with each other during these weeks, write your email address in the Canvas chat. Check out.	Feedback on action plans is a form of *reinforcement.* Support from and discussions in the group contributes to *reciprocal determinism* and *observational learning.*	To revise and formulate an action plan can be part of *problem‐solving, decision‐making and self‐tailoring*.

Home assignment to be completed after Session 6		
Purpose: Continue to use the action planning process and, if appropriate, make a new action plan		
Content	Social Cognitive Theory	Lorig and Holman tasks and skills
Carry out the planned fall preventive activities/strategies. Self‐evaluation: What fall preventive strategies work well? How can you maintain these strategies? Review your action plan regularly and make a new action plan if you identify new fall risks. If wanted, use the Canvas chat.	Internal feedback/evaluation can contribute to *reinforcement*. *If one succeeds* = *behavioural capability*, which leads to *self‐efficacy*. Expectations and thoughts about the future influence *outcome expectations.*	To carry out the activities implies *taking action,* and to self‐evaluate supports *problem‐solving* and *decision‐making.*

Session 7		
Purpose: Gathering and conclusion as well as repetition of goals, behaviour change and motivation		
Content	Social Cognitive Theory	Lorig and Holman tasks and skills
Check in. The session's purpose is presented. Homework follow‐up. Mini‐lecture/discussion: Rehearsal (from Sessions 3 and 6): goals and motivation and different phases in behaviour change, as well as supportive and motivating factors. Social support onwards. Who can you ask for help and support? Group networking after this programme? Check out: Lessons learned from the programme. Feel free to share your experiences from the programme or something else you want to share.	To hear about each other's results = *observational learning.* Feedback on the activities can promote *reinforcement.* Talking about what you have managed to do strengthens *behavioural capability*, which can lead to increased *self‐efficacy.*	To reflect on whom to ask for help from provides opportunities for *resource utilization.*

*Note:* Social Cognitive Theory (SCT): The core components included in the SCT were as follows: reciprocal determinism, behaviour capability, observational learning, reinforcement, outcome expectations and self‐efficacy. Lorig and Holman tasks: medical, role and emotional management; Lorig and Holman skills: problem‐solving, decision‐making, resource utilization, patient–provider partnership, action planning, self‐tailoring and taking action.

### 2.7 Train‐the‐Trainer Sessions and Programme Material Development

In the train‐the‐trainer sessions, two members of the operative group (M.F. and C.Y.) applied the pedagogical method of flipped classroom [31, 32] to train the group leaders. The group leaders were healthcare professionals recruited to serve as facilitators for an entire *Fewer Falls in MS* cycle (i.e., seven sessions) in the Beta test of the *Fewer Falls in MS* programme in December 2021. Two physiotherapists and one nurse were recruited by the research group via their professional network to serve as group leaders. The group leaders had expertise in neurological care, neurological rehabilitation and fall prevention. Before the train‐the‐trainer sessions, the group leaders were supposed to have read the PowerPoint presentations for the train‐the‐trainer sessions and the manual (developed by the research group for the group leader's facilitation). During the three 2‐h train‐the‐trainer sessions, the group leaders got acquainted with and discussed theories, models, manual, programme material and approaches to use as a group leader (e.g., to create an open, safe and stimulating group atmosphere and to acknowledge individual group members). The group leaders also got the opportunity to be confident in using the digital platforms Zoom (Zoom Video Communications, Inc.) and the online learning platform Canvas (Canvas, Instructure Inc.). The three train‐the‐trainer sessions contained: theoretical briefings, role plays and refinements of the PowerPoint presentations. The three group leaders were encouraged and supported to make the manual their own, that is, change expressions, and make comments in the text.

### 2.8 Programme Testing

In January and February 2021, a ready‐for‐testing (i.e., Beta) version of the programme was pretested. This Beta version of the *Fewer Falls in MS* intervention included a total of seven sessions: six sessions occurring weekly and one booster session occurring 6 weeks after the sixth session. However, due to time constraints during this practical pretest of the programme, the six sessions occurred twice weekly, and the seventh session was not performed. Four of the pwMS, three ambulatory and one non‐ambulatory, who had participated in the co‐design and co‐development phases were recruited to serve as programme participants. The aim of the practical pretest was twofold: to explore how the intervention worked in practice and to serve as education and practice of the group leaders. To give all three group leaders the possibility to practice programme delivery, each of the group leaders delivered two programme sessions each. The different pedagogical tools applied were the use of short films, PowerPoint presentations and a chat using the online learning platform Canvas. The six synchronous online intervention sessions were delivered via Zoom video conferencing and included brief didactic presentations by the group leaders, whole group discussions and small group discussions.

The researchers in the operative group attended each of the six Beta test sessions to provide technical support, take field notes and answer participant's questions regarding home assignments. Separate dialogues with each of the group leaders were offered by the researchers before each session to sort out any questions or problems related to the Zoom and/or Canvas usage. After each session, the researchers conducted a follow‐up dialogue with the pwMS serving as programme participants and the group leaders to identify opportunities to improve content or processes.

## 3 Results

### 3.1 Scoping Review

The scoping review identified 14 publications in total [19]. Ten articles represented six different fall prevention interventions. Briefly, three of the publications were randomized controlled trials with relatively small sample sizes [16, 17, 18], whereof one also included people with other neurological conditions [17]. The interventions tested in the trials did not favour intervention or control groups; so, more evaluations of self‐management fall prevention interventions are needed. None of the 14 publications included a self‐management definition, and self‐management content was variable and not comprehensive in nature. The few intervention studies showed that the research on self‐management of falls in pwMS is in its infancy. The results from the scoping review highlighted the importance of using a clear definition of self‐management and supported the plan to develop a self‐management fall prevention programme for both ambulatory and non‐ambulatory pwMS.

### 3.2 Co‐Development

The co‐development together with patient organization Neuro Sweden gave the researchers in the operative group essential contributions regarding for example length of co‐design sessions, number of pauses during each session and recruitment of participants for the workshops. Later in the co‐development phase, the research group received input from pwMS and healthcare professionals on programme content and format details, which were used to refine and modify the programme. As an example, pwMS suggested simplifications of the goal‐setting element of the programme and improved the layout of the action‐plan template. The co‐development process was stretched over a period of 23 months. For each meeting with pwMS, healthcare professionals and the research group, respectively, steps were successively taken in the programme's development. The relatively long‐time perspective provided conditions for continuous feedback and dialogue with all stakeholders during the process and provided opportunities for reflection and reconsideration on the programme details (see below).

### 3.3 Co‐Design Workshops

Within the co‐design process, six *ideas and correlating needs* emerged, here presented in general, and detailed data and analyses are presented elsewhere [33]. The content formed an important basis that was considered in the continuing development process:

1. *Understanding one's fall risks*: The participants wanted the programme to assist them in identifying their needs and fall risks. They described that as MS is a progressing disease, it involves an emerging loss of abilities and threats to lose abilities.
2. *Being in control over one's own life*: The participants described a need to feel in control over their situation. In relation to fall prevention, this concerned the ability to predict events that could lead to a fall and make plans to prevent this. But it is also related to strengthening the self‐esteem and feel that they could control their own life.
3. *Using mobility aids*: The participants suggested that the programme should include discussions about using mobility aids that include pros and cons of using aids and consequences of not using mobility aids. The participants recommended practical training on how to use aids and suggested that discussions about mobility aids with peers could diminish the feeling of being stigmatized when using mobility aids.
4. *Keeping the individual perspective during group meetings*: The participants wanted a group‐based programme that addressed the shared needs of the group while also addressing the unique needs of individual group members because fall risks are heavily dependent upon the individual's situation.
5. *Managing stress*: The participants described the need for content on stress management that addressed internal sources of stress (e.g., unrealistic expectations about physical and mental coping) and external sources of stress (e.g., social views and expectations).
6. *Training to improve balance*: The participants suggested that the programme should include balance training and opportunities to increase physical activity. They wanted practical training to prevent falls during daily activities, such as maintaining balance while reaching for something on a shelf in the kitchen.

The workshops with pwMS also yielded important insights into participants' preferences about programme processes. For example, the pwMS who participated in the co‐design workshops did not want relatives to participate in the programme because it would interfere with the group process and shift focus away from the pwMS. Further, online delivery would be suitable because it would make it possible to participate in the programme even when energy levels were low.

### 3.4 Identification of Guiding Theories, Approaches and Pedagogical Models

The research group decided to use Lorig and Holman's operationalization of self‐management to guide the programme content [30]. Lorig and Holman were among the first to operationalize the self‐management approach. The three self‐management tasks (medical management, role management and emotional management) and six self‐management skills (problem‐solving, decision‐making, resource utilization, the formation of patient–provider partnership, action planning and self‐tailoring) were incorporated into *Fewer Falls in MS* content.

Lorig and Holman [30] describe self‐efficacy as one of the possible mechanisms by which self‐management is achieved. Self‐efficacy is one of the core components in Social Cognitive Theory (SCT) [34], and it was identified as the leading theory to guide development of *Fewer Falls in MS* content. The SCT attention to learning from others was well aligned with the programme's focus and group format. The following components are essential in SCT and were considered relevant for the emerging programme (see Table 1):

1. *Reciprocal determinism* is the central concept of SCT and refers to the dynamic and reciprocal interaction of person (with learned experiences), environment (external social context) and behaviour (responses to stimuli to achieve goals).

2. *Behavioural capability* refers to a person's ability to perform a behaviour through essential knowledge and skills.
3. *Observational learning* asserts that people can observe a behaviour conducted by others and then reproduce those actions.
4. *Reinforcements* refers to the internal or external responses to a person's behaviour that affect the likelihood of continuing or discontinuing the behaviour.
5. *Expectations* refers to a person's anticipated consequences of own behaviour. Outcome expectations derive largely from previous experience.
6. *Self‐efficacy* refers to the level of a person's confidence in his or her ability to successfully perform a behaviour in a specified situation.

Pedagogical models describing behavioural change were identified through search in the literature and through discussions within the research group. Two overarching pedagogical models were identified: *a blended learning design* [32, 35] and *the universal design for learning* [36]. The blended learning design was found suitable for an online programme as it describes synchronous online group learning sessions and asynchronous home assignments [32]. Meyer's universal design for learning was also found suitable for the programme's intentions as it emphasizes that an intervention should enable participants of various abilities to assimilate the knowledge using different forms of engagement, materials, action and expression [36].

### 3.5 Programme Format and Content

The development process resulted in a core structure for the *Fewer Falls in MS* programme, that is, six 2‐h online sessions with mini‐lectures and group discussions, plus one booster session. Home assignment activities occur after each of the six sessions, and findings from home assignment activities serve as basis for whole group conversations.

The research group decided that online delivery of the programme was optimal since it was supported by the pwMS in the co‐design workshops and by the literature [37].

The results stemming from the co‐design process, the discussions within the research group and with stakeholders and the application of selected theories, approaches and models, all led to determining the following priorities for the intervention: (1) to identify one's own fall risk situations; (2) to make one's own action plan and follow out the planned activities; (3) to be aware of one's own capacity in relations to expectations and demands in daily life; (4) to follow‐up on the action plan and (5) to learn about behavioural change and maintenance of motivation. The programme content and the action plan layout were developed using input from international colleagues, see Acknowledgements. Details of the programme are displayed in Table 1.

### 3.6 Practical Tests

During the train‐the‐trainer sessions, some challenges associated with online delivery were identified. For example, group leaders expressed difficulty connecting with the participants and show empathy, while simultaneously focussing on the technical aspects of the Zoom platform. To address these challenges, additional technique training in the use of the Zoom platform was offered to the group leaders. The experiences and lessons learned during the train‐the‐trainer sessions also led to minor programme revisions (e.g., revisions of PowerPoint presentations and group leaders' manual).

During the Beta testing of the programme, feedback from the pwMS participants and the group leaders involved in each session led to several improvements. Specifically, the goal‐setting activities were simplified, and the action plan template was improved. Additionally, importance of providing group leaders with a quiet room and a well‐functioning computer was identified.

The entire development process resulted in the version of the *Fewer Falls in MS* intervention that will be subjected to feasibility testing [38].

## 4 Discussion

The *Fewer Falls in MS* programme is unique among fall prevention programmes for pwMS as the programme's intended end users include non‐ambulatory as well as ambulatory pwMS. Additionally, the programme resulted from an iterative co‐development process that involved healthcare professionals and other representatives of its target audience: ambulatory and non‐ambulatory pwMS. The development process resulted in an online, group‐based programme that aims to reduce fall incidence among both ambulatory and non‐ambulatory pwMS through utilization of a carefully operationalized self‐management approach.

The programme development process had several strengths. Specifically, the programme was developed to address the lack of interventions for pwMS that utilize self‐management approaches to target self‐managing environmental, psychological and behavioural influences on fall risk regardless of ambulation level, that is, for both ambulatory and non‐ambulatory pwMS. Consistent with the recommendations made by Skivington et al. [24], stakeholders were involved throughout the whole development process. The co‐design process led to a deep understanding of the fall prevention needs of pwMS. As one example, creating content designed to help participants predict and manage situations that could increase the risk for a fall during daily activities was identified as a priority.

Adherence to the MRC guidelines [23, 24] was an additional strength of the development process. Those guidelines highlighted the importance of using theory to describe expected mechanisms of the programme and informed the process of developing and refining the intervention. Our development process was iterative in nature, involved different phases occurring concurrently and Beta testing. The MRC guidelines also emphasize the importance of reusing existing programmes to minimize research waste. The research group used the *Safe at Home BAASE* programme developed and evaluated by Finlayson et al. [13] as the starting point of departure for the development of the *Fewer Falls in MS* programme but recognized an important opportunity to further develop application of self‐management approach and theoretical models. The idea to further develop the self‐management approach was based on recent research on the importance of strengthening self‐management in people living with chronic conditions [11, 39] and on the research group's clinical and research experience on fall prevention for pwMS. Both the self‐management and SCT features were carefully mapped to the intervention, and the resulting resources (Table 1) will be valuable tools for group leaders to support programme fidelity.

Initial planning for *Fewer Falls in MS* did not include a definitive decision to develop an online programme; however, the COVID‐19 pandemic led to new conditions that demanded digital delivery. Online delivery of *Fewer Falls in MS* was supported by literature, which pointed to the feasibility of group‐based interventions delivered online and additional advantages, including improved programme reach and lower costs due to the elimination of travel expenses [37]. The digital delivery of the programme was aligned with the pedagogical models identified and enthusiastically supported by the pwMS involved in the co‐design process. Despite the recognized advantages, some challenges associated with the online format were observed during our train‐the‐trainer sessions and the practical pretest sessions. For example, programme participants and group leaders alike experienced technical challenges, and group leaders spoke to the difficulty to focus both on the individual participants while managing the digital platform. Similar difficulties associated with online programme delivery have been identified in the literature [37]: the participants may experience technical difficulties (e.g., loss of internet connection), and social interactions may be compromised due to the lack of direct visual contact with the participants. Collectively, our experiences during the development process regarding online delivery resonated with the conclusion by Branbury et al. [37] that online delivery is feasible and has the potential to improve accessibility.

Although many of the pwMS involved in the co‐design process made a recommendation to include balance exercises and hands‐on training in use of mobility aids in *Fewer Falls in MS*, we did not act on that recommendation primarily because our overarching objective was to create a fall prevention self‐management programme for pwMS. This self‐management approach afforded programme participants with opportunities to address balance training or mobility aid needs through individual action plans. We also recognized that the ambulatory and non‐ambulatory individuals participating in *Fewer Falls in MS* had very different balance training and mobility aid needs. The time required to adequately address these topics, if they were to be a focus of the programme, would be substantial and alter our original priority for the programme, which was to facilitate participants' self‐management of fall risk. Overall, the carefully operationalized self‐management approach is a central feature of *Fewer Falls in MS.* It is interesting to note that while *Fewer Falls in MS* and *Safe at Home BASE* are similar in their application of social learning theory and diverse influences on fall risk, the content of the two programmes is distinctly different. Although increasing knowledge of the connections between MS symptom management (e.g., loss of balance, fatigue) and falls prevention was a priority for the *Safe at Home BASE* programme, *Fewer Falls in MS* dedicates more time to the process of creating, enacting and following up on individual action plans.

This study had methodological limitations to consider. In the development of a complex intervention, deep understanding of the problem is essential [20, 26]. The scoping review [19] showed that only a few qualitative self‐management studies on fall prevention for pwMS have been published. An initial thorough exploration, with a qualitative approach, on how pwMS uses self‐management strategies to prevent falls would have further contributed to a rich understanding of the problem. However, the issue was explored to some extent in one of the co‐design workshops with pwMS [33]. An additional limitation to consider is control over the environment in which the programme was delivered [20]. The researchers did not plan the group leaders' programme delivery environment. Clear instructions to group leaders about the importance of preparing the room and computer well in advance of the sessions may have helped to prevent technical challenges and the associated stress for group leaders.

Based on the literature, a process for a well‐developed complex intervention ends with a feasibility testing phase [20]. Therefore, this online self‐management fall prevention programme, developed in a systematic and iterative process in cooperation with ambulatory and non‐ambulatory pwMS, will be evaluated in a feasibility study. A study protocol on the feasibility study has been published [38].

## 5 Conclusion

A carefully operationalized definition of self‐management and an iterative co‐development process were essential to the creation of the *Fewer Falls in MS* programme. Continuation of the co‐development process was needed to refine the programme. The theoretical underpinnings of the intervention, self‐management approach and findings from the co‐development process will inform process and outcome evaluation priorities.

## Author Contributions

**Susanna Tuvemo Johnson:** conceptualization, methodology, data curation, investigation, formal analysis, writing – original draft, writing – review and editing. **Charlotte Ytterberg:** conceptualization, investigation, funding acquisition, writing – original draft, methodology, writing – review and editing, project administration, formal analysis, data curation. **Elizabeth Peterson:** conceptualization, funding acquisition, writing – review and editing. **Sverker Johansson:** conceptualization, funding acquisition, writing – review and editing. **Marie Kierkegaard:** conceptualization, methodology, data curation, investigation, formal analysis, writing – review and editing, funding acquisition. **Kristina Gottberg:** conceptualization, methodology, data curation, investigation, formal analysis, funding acquisition, writing – review and editing. **Maria Flink:** conceptualization, methodology, data curation, investigation, formal analysis, funding acquisition, writing – original draft, writing – review and editing.

## Ethics Statement

Ethical approval was obtained from the Swedish Ethical Review Authority (Dnr 2019‐06030 and 2020‐04286).

## Consent

Oral and written consent was obtained from all participants.

## Conflicts of Interest

Marie Kierkegaard has received honoraria for lectures from Novartis, Sanofi Genzyme and Merck.

## Data Availability

The data sets generated and/or analysed during the current study are not publicly available but can be available upon reasonable request. As data can indirectly be traced back to the study participants, according to the Swedish and EU personal data sharing legislation, access can only be granted upon request. Request for access to the data can be put to our Research Data Office (rdo@ki.se), Karolinska Institutet and will be handled according to the relevant legislation. In most cases, this will require a data processing agreement or similar with the recipient of the data.
